# Preclinical modeling and multimodality imaging of chronic myocardial infarction in minipigs induced by novel interventional embolization technique

**DOI:** 10.1186/s13550-016-0214-7

**Published:** 2016-07-08

**Authors:** Bo Tao, Haokao Gao, Minwen Zheng, Zhonghua Luo, Liwen Liu, Wei Bai, Jing Wang, Daliang Liu, Sai Ma, Zhenli Luo, Lei Gao, Yabin Wang, Feng Cao

**Affiliations:** Department of Cardiology, Chinese PLA General Hospital, Fuxing Street 28#, Haidian District, Beijing, 100853 China; Department of Cardiology, Xijing Hospital, Fourth Military Medical University, Xi’an, 710032 China; Department of Radiology, Xijing Hospital, Fourth Military Medical University, Xi’an, 710032 China; Department of Ultrasonography, Xijing Hospital, Fourth Military Medical University, Xi’an, 710032 China; Department of Nuclear Medicine, Xijing Hospital, Fourth Military Medical University, Xi’an, 710032 China

**Keywords:** Pig model, Myocardial infarction, Percutaneous coronary artery embolization, Balloon, Sponge

## Abstract

**Background:**

This study was designed to establish a chronic myocardial infarction (MI) model in minipigs with a novel coronary sequential balloons-sponge embolism technique.

**Methods:**

Eighteen healthy minipigs (25–30 kg) were randomly divided into three groups for left anterior descending artery (LAD) occlusion: conventional balloon occlusion group (BO group, temporary balloon occlusion for 60 mins), half-balloon embolism group (HB group), and sequential balloon-balloon-sponge embolism group (BBS group, two half-balloons with one sponge as the embolism clot). The incidence of ventricular fibrillation (VF), total mortality, operating time, and vascular recanalization 3 months post-MI was recorded and compared. Echocardiography, multimodality nuclear medical imaging, and histology staining were applied for the evaluation of infarction.

**Results:**

Thirteen out of 18 minipigs survived after the operation, while 5 animals died with VF (3 in the BO group, 1 in the HB group, and 1 in the BBS group), with an 83.3 % (5/6 minipigs) acute procedural survival rate in embolism groups. The operating duration was 60.0 ± 0.5 mins, 21.4 ± 5.2 mins, and 31.2 ± 4.7 mins in the three groups, respectively. LAD recanalization was found in three animals of the HB group but none in the BBS group by angiography follow-up. The infarct sizes were more stable and larger in the HB group and BBS group than that in the BO group (*P* < 0.05, *n* = 13).

**Conclusions:**

The method of sequential balloons-sponge embolization could induce myocardial infarction with consistent and sustained embolization and gain higher operation success rate and better repeatability in minipigs, which holds a promising method for preclinical MI study.

**Electronic supplementary material:**

The online version of this article (doi:10.1186/s13550-016-0214-7) contains supplementary material, which is available to authorized users.

## Background

Coronary artery disease and subsequent myocardial infarction (MI) are leading causes of morbidity and mortality in the world [1, 2]. Although advances in MI therapy have improved acute survival rates, such as percutaneous coronary intervention or coronary artery bypass graft, survivors are prone to development of chronic degenerative changes as a result of irreversible cardiomyocyte loss, extracellular matrix fibrosis, and pathological gene programming [3, 4]. Large animal model of chronic MI with long-term postoperative survival is very important for studying the molecular and cellular changes following MI and testing novel therapies for prevention of chronic negative myocardial remodeling.

At present, MI modeling in most of preclinical experiments still apply surgical opening chest and coronary artery ligation or incision of the peripheral arteries for interventional approach and balloon occlusion of coronary flow, which are also supported by endotracheal intubation and assisted respiration. Left anterior descending coronary artery (LAD), especially mid-LAD was used for target artery [5–8]. The biggest advantage is that they maintain the consistency of infarct size and target vessel status. However, some issues and potential pitfalls should be recognized such as high incidence rate of malignant ventricular arrhythmias, less induced infarct size, longer operation time (generally required 5–6 h), and larger trauma. In this operation course, the blood flow interrupts completely and suddenly, resulting in a high incidence rate of malignant ventricular arrhythmias and mortality [9, 10]. The temporary blockage of blood flow by balloon usually results in less induced infarct size and fast revascularization afterward, which is apt to overestimate the infarct size in the early stage [11]. In recent years, many methods of coronary artery embolization have been proposed and applied in large animals’ MI modeling. But to our knowledge, these methods still need further optimization, due to limitations in high cost (spring coil) [12], difficulty in pinpointing embolism levels (micro-catheter injection of ethanol, micro-spheres embolization) [13–16], poor operational feasibility (sponge is hard to push) [17], and postoperative vascular recanalization (autologous blood clots, winding stent, spring coil) [18–20].

In the present study, we initiated a consistent and sustained coronary embolism model with high operation success rate and short operative time by the sequential balloons-sponge embolization in a large animal so as to lay a good foundation for further heart intervention. Furthermore, multimodality clinical imaging and histology staining were applied to evaluate the efficacy of the method compared with traditional modeling by interventional technology.

## Methods

### Experimental protocols

This study was performed at an Association for the Assessment and Accreditation of Laboratory Animal Care International-accredited large animal research facility. All animal procedures were conducted in conformity with the National Institutes of Health Guide for the Care and Use of Laboratory Animals. A total of 18 minipigs (female, weighing 25–30 kg) were randomly allocated into three groups (*n* = 6, each group) including balloon occlusion group (BO group), half-balloon embolism group (HB group), and sequential balloon-balloon-sponge embolism group (BBS group, two half-balloons with one sponge as the embolism clot). Coronary artery occlusion or embolization was kept in the mid-LAD, immediately after the ostium of the second diagonal branch [5, 10, 21, 22]. The following data were compared in three groups: the incidence of ventricular fibrillation (VF), total mortality, operation time of occlusion or embolization, and vascular recanalization at 3 months post-MI. MI in the BBS group was confirmed by electrocardiogram, echocardiography, and ^18^F-FDG positron emission tomography/computed tomography (PET/CT) imaging. The combination evaluating methods of echocardiography, single photon emission computed tomography/computed tomography (SPECT/CT), and histology staining were applied to compare the infarct size in three groups.

### Common procedures of MI induction in three groups

The three groups all went through the following six procedures including anesthesia, percutaneous femoral artery puncture, selective coronary angiography (CAG), conventional ischemic preconditioning and prevention of arrhythmia, balloon occlusion or embolization, and postoperative monitoring and treatment.

**Fig. 1:**
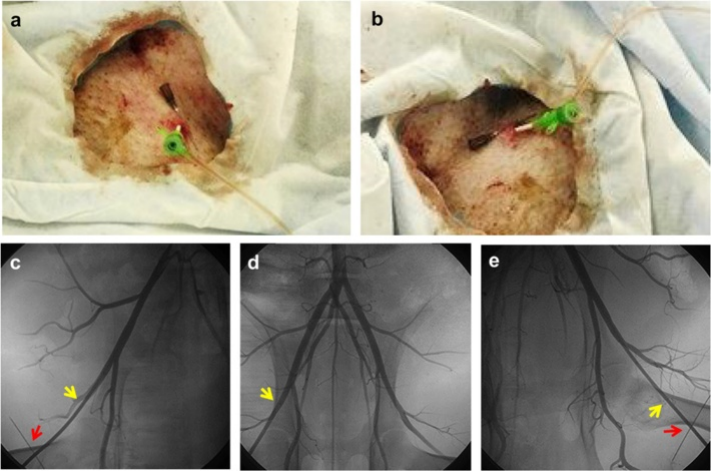
Minipig femoral artery angiography in three groups. **a**, **b** Display arterial introducer sheath in the right and left femoral artery, respectively. **c**–**e** The angiography of right and left femoral artery and iliac artery, internal iliac artery, and abdominal aorta, respectively. The *yellow arrows* indicate the location of introducer sheath, and the *red arrows* indicate the location of skin puncture

Briefly, animals were anesthetized with ketamine (20 mg/kg, IM) and introduced intravenous catheters in marginal ear veins. Continuous infusion of propofol (0.003 ml/kg/min) was administrated to maintain anesthesia. Animals were continuously monitored and inhaled oxygen (3 L/min). The right or left femoral artery was identified, and a 6-Fr radial arterial introducer sheath (Terumo, Japan) was advanced over the guidewire in the femoral artery by percutaneous puncture. When it was necessary, ultrasound could be used to assist the puncture. Heparin sodium was given as a 150 IU/kg bolus IV at the start of the procedure and continued at 50 IU/kg/h IV throughout the operation. Femoral artery angiography was performed (Fig. 1), and a 6-Fr guiding catheter (Judkins right 4.0 type) was advanced into the ostium of the coronary artery. Baseline selective coronary angiography and left ventricular angiography were performed, and the target blocking location was determined. Lidocaine (0.03 mg/kg/min) was intravenously infused throughout the following procedures. In case of VF, nonsynchronized direct current defibrillation was performed at 360 J. Along with the guidewire, the 1.5–2.5-mm balloon catheter according to target vessel diameter was inflated and deflated three times in the mid-LAD as the ischemic preconditioning [23, 24]. Each time, the balloon was inflated 6–8 atm for 5 mins with the interval of 1 min. Subsequently, all animals were performed mid-LAD balloon occlusion or embolism procedure according to grouping. Finally, the arterial sheath was removed, followed by 30 mins oppression on the femoral artery puncture area. After the procedures, all the animals received antibiotics (penicillin 640 WU once a day, for 3 days).

### Procedures of balloon occlusion or embolization

#### Balloon occlusion group (Classic method of MI induction)

**Fig. 2:**
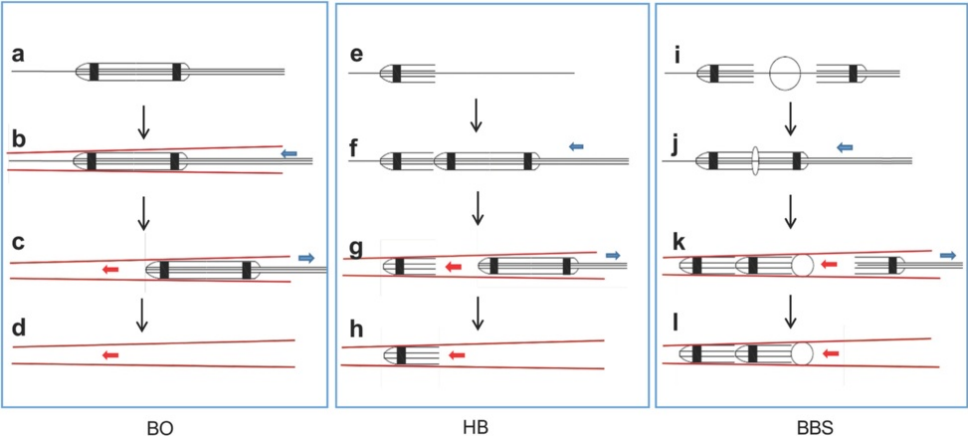
Pattern graphs of procedures in three groups. **a**–**d** Balloon occlusion group. **e**–**h** Half-balloon embolism group. **e**–**l** Sequential balloons-sponge embolism group. The *blue arrows* indicate the direction of the balloon catheter, and the *red arrows* indicate the direction of the blood flow

**Fig. 3:**
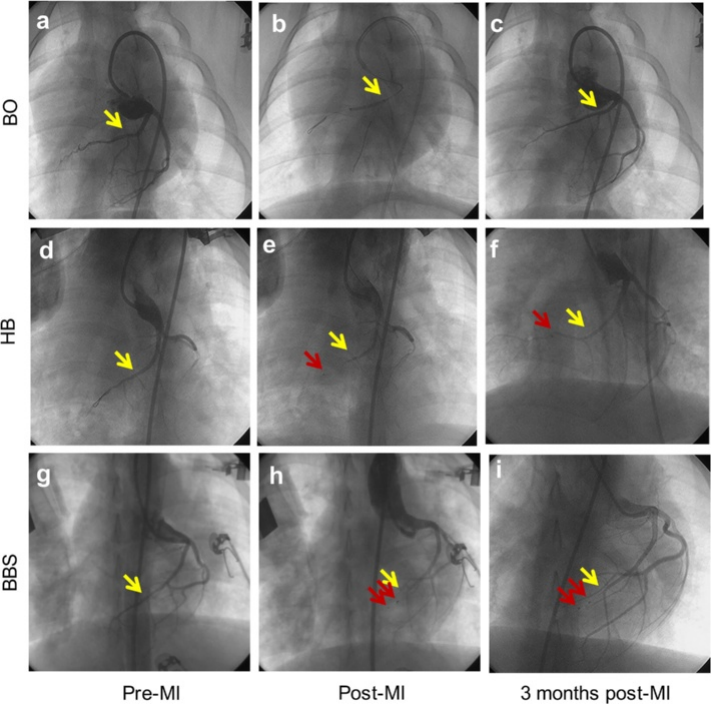
Presentation and comparison of key operation procedures in three groups. **a**, **d**, **g** Selective coronary angiography determined the location of balloon occlusion or embolism in three groups. **b**, **e**, **h** Blood flow was completely blocked after balloon occlusion or embolism of the distal LAD coronary artery in three groups, and the blood flow of the second diagonal branch was still normal and clear. **c**, **f**, **i** Three months after operation, coronary angiography confirmed that three minipigs have vascular recanalization in the HB group; meanwhile, no vascular recanalization occurred in the BBS group. The *yellow arrow* indicates the location of the second diagonal branch, and the *red arrows* indicate the location of the metal marker of balloon

After ischemic preconditioning, matched angioplasty balloon (with the diameter of targeted artery position) was advanced to the mid-LAD. The balloon was inflated at 6–8 atm for 60 mins to block the blood flow completely without affecting the second diagonal branch. After that, the balloon was deflated and withdrawn, and LAD was reperfused (Figs. 2a–d and 3a–c).

#### Half-balloon embolism group

After ischemic preconditioning, two balloon catheters were used. The first inflated balloon was cut in the median, and the distal free half-balloon (about 10 mm, half spindle type, withered shape, with the tip of a metal marker) was set along the guidewire. After that, the second balloon catheter was set along the guidewire and pushed 10 mm distal to the ostium of the second branch of LAD. Then, the guidewire was retraced to the second balloon catheter. During this process, there was no displacement in the distal half-balloon (with the metal marker that can be seen in fluoroscopy). After that, the guidewire and the second balloon catheter were withdrawn from the coronary artery. Intermittent coronary angiography was performed, and the time of the complete interruption of blood flow was recorded (Figs. 2e–h and 3d–f).

#### Sequential balloons-sponge embolism group

Apart from two balloon catheters, a sponge block (compressible, nonbiodegradable, sterilized, with good biocompatibility) was prepared. The first half-balloon was pushed to 20 mm distal to the ostium of the second branch of LAD according to the method above. After 10–20 mins, the blood flow of LAD was diminished and completely interrupted. During this time, the second half-balloon was cut and prepared. In turn, the second free half-balloon and 1.5–2.5 mm sponge block and half-balloon with a catheter (similar to a sandwich structure) were set along the guidewire and pushed to 10 mm distal to the ostium of the second branch of LAD. Following angiography and accurate positioning, the guidewire was withdrawn to the second balloon catheter. During this process, there was no displacement in the two free half-balloon and sponge. After that, the guidewire and the second balloon catheter were withdrawn from the coronary artery. The time of the complete interruption of blood flow was recorded (Figs. 2e–l and 3g–i).

### Coronary angiography and left ventricular angiography follow-up at 3 months post-MI

Three months after MI, all survived minipigs were subjected to coronary angiography and left ventricular angiography to observe the blood flow condition of LAD and the heart function. Pigtail catheter and high-pressure injector were applied. The procedures were the same as previous procedures.

### Evaluation of electrocardiogram, echocardiogram, nuclear medical imaging, and histology staining

Electrocardiogram (ECG) and ultrasonic cardiogram (UCG) were performed prior, immediately, 1 month, and 3 months postoperation. ECG-gated SPECT/CT imaging was performed 1 week before MI and 3 months after MI with a system (SymbiaT2, Siemens, Germany) in three groups. Myocardial perfusion images acquired 1 week before MI was taken as baseline data. Myocardial perfusion images were acquired and semi-quantitative assessment of left ventricular ejection fraction (LVEF) and total perfusion deficit (TPD) were processed as described in our previous study [25]. ^18^F-FDG PET/CT imaging was performed 1 week before MI and 3 months post-MI in BBS group with an available system (Biograph40, Siemens) to confirm the formation of myocardial infarction. Histological analysis was performed 3 months post-MI. These classic methods have been fully described by previous investigators and introduced in our Additional file 1 [26, 27].

### Statistical analysis

SPSS14.0 (Chicago, USA) was used for data analysis. To identify significant differences in mortality, incidence of VF, and vascular recanalization among the three groups, Fisher exact test was applied. Continuous variables that approximated the normal distribution were expressed as mean ± standard error (SEM). Multiple group comparisons were performed by one-way analysis of variance (ANOVA) followed by the least significant difference (LSD) *t* test for post hoc analysis. Comparisons between the two independent groups were analyzed using the Student’s *t* test. *P* < 0.05 was considered statistically significant.

## Results

### Viability and myocardial infarction reproducibility

**Fig. 4:**
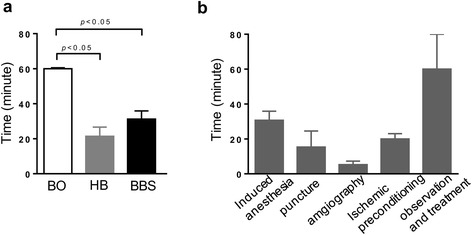
Comparison of operation time. **a** Balloon blocking or embolism time in three groups. **b** The mean time of each operation procedure in three groups

Eighteen minipigs all underwent the procedures, while 5 animals died as a result of intractable VF (3 animals of the BO group, 1 in the HB group, and 1 in the BBS group) with an 83.3 % (5/6 pigs) acute procedural survival rate in the HB group and the BBS group. Incidence of VF and total mortality in the HB group and BBS group was markedly reduced as compared with the BO group (*P* < 0.05, *n* = 18). Balloon blocking or embolism time was 60.0 ± 0.5, 21.4 ± 5.2, and 31.2 ± 4.7 mins in the BO group, HB group, and BBS group, respectively (*P* < 0.05) (Fig. 4a). In addition, percutaneous femoral artery puncture was performed for a total of 31 times in this study with the operation time around 15.4 ± 6.2 mins. Ultrasound assistance was used once only. The mean time of each procedure in the operation of three groups was presented in Fig. 4b. In general, the entire procedure of each minipig can be achieved within 2–3 h. The follow-up of coronary angiography 3 months after operation revealed that three minipigs in the HB group appeared to have vascular recanalization, meanwhile none happened in the BBS group (*P* < 0.05, *n* = 10).

### Confirmation of myocardial infarction by ECG, UCG, and ^18^F-FDG PET/CT imaging

**Fig. 5:**
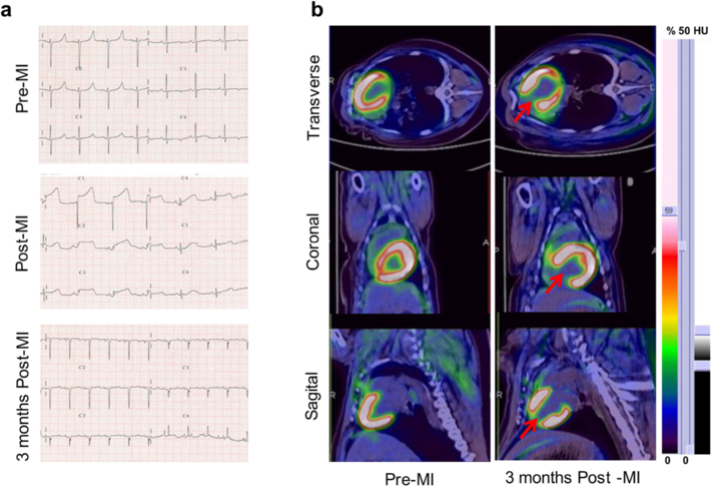
Confirmation of myocardial infarction by ECG and ^18^F-FDG PET/CT imaging. **a** ECG confirmed ST segment of the anterior wall leads dynamic changes in different time points. **b** Hybrid imaging of myocardial ^18^F-FDG PET/CT showed that the apical level and anterior wall and anterior septal of mid-level in the BBS group presented myocardial metabolic defect 3 months post-MI. The average standard uptake value (SUV) of region of interest (ROI) indicated by *red arrows* was 1.50 ± 0.52, which was lower than 8.24 ± 1.29 with SUVavg of non-ROI (*P* < 0.05, *n* = 5)

**Fig. 6:**
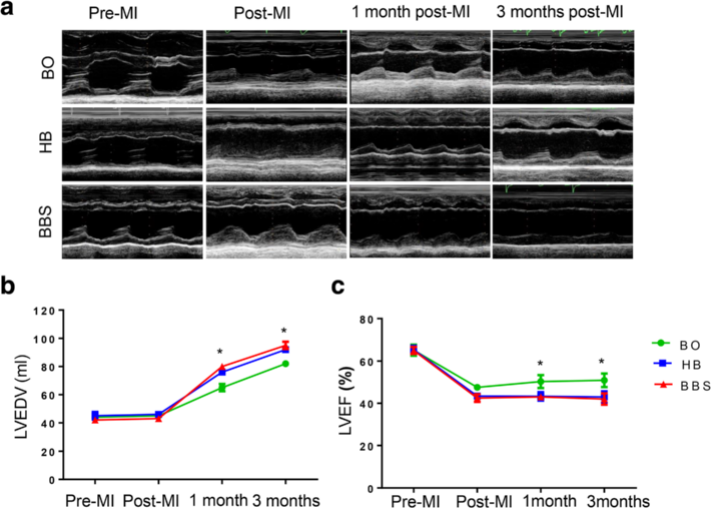
Echocardiographic evaluation of cardiac function. **a** Representative M-mode echocardiographic data of hearts at different time points in three groups. The dynamic changes of myocardial infarction in the BO group were only observed in the apical level, and MI of the HB group and BBS group could be observed in the mid-level of the left ventricle. **b**, **c** Echocardiographic evaluation of cardiac LVEF and LVEDV (*P < 0.05)

The dynamic ST-segment elevation of the anterior wall leads was observed during the procedure (Fig. 5a). ^18^F-FDG PET/CT imaging showed that myocardial metabolic defect was presented in the myocardium of apical level, anterior wall, and anterior septal in the BBS group (Fig. 5b). Baseline LVEF and the left ventricular end-diastolic volumes (LVEDV) values in UCG were equivalent in the three groups. After MI, there was a significant reduction in LVEF, which is different among the three groups. The LVEF of the BO group at pre-MI, immediately post-MI, 1 month, and 3 months post-MI were 65.2 ± 2.6, 47.6 ± 1.4, 52.3 ± 3.1, and 52.9 ± 3.2 %. The values of LVEF in the HB group at the same time points were 65.4 ± 1.8, 43.4 ± 1.2, 43.3 ± 2.0, and 43.0 ± 2.7 %, respectively. And the LVEF in the BBS group were 65.0 ± 1.7, 42.5 ± 1.8, 43.0 ± 1.2, and 42.0 ± 2.7 % at pre-MI, immediately post-MI, 1 month, and 3 months post-MI. The LVEF in the HB group and BBS group was significantly decreased at 1 month, and 3 months post-MI as compared with the BO group (*P* < 0.05). Meanwhile, the LVEDV in the HB group and BBS group increased higher than that in the BO group (76.0 ± 2.0 and 80.0 ± 2.2 vs 65.0 ± 2.7 at 1 month post-MI, *P* < 0.05; 92.0 ± 1.7 and 95.0 ± 2.7 vs 82.0 ± 2.2 at 3 months post-MI, *P* < 0.05). There was no significant difference in LVEF or LVEDV between the HB group and BBS group (*P* > 0.05) (Fig. 6a–c). Two movies of the left ventricular angiography in the BBS group at pre-MI and 3 months post-MI were provided in Additional files 2 and 3, respectively.

### Lowest myocardial perfusion in BBS group

**Fig. 7:**
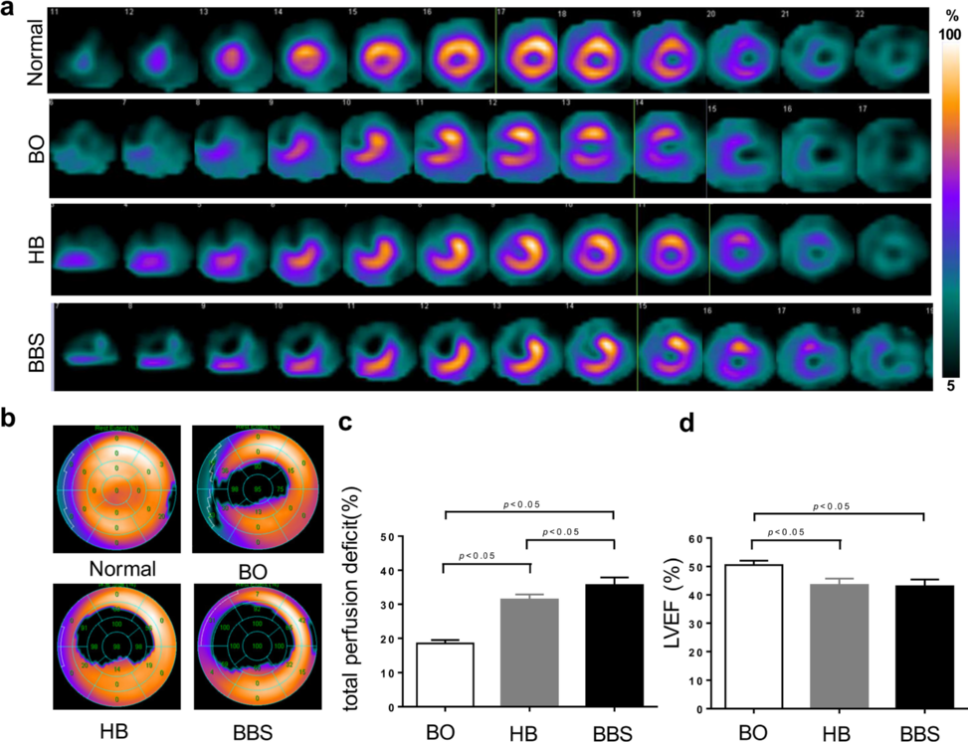
Myocardial perfusion by SPECT/CT imaging. **a**, **b** Representative short axis slices and Polar bull’s eye plot of normal myocardial perfusion and perfusion defect in three groups 3 months post-MI. **c**, **d** Semi-quantitative assessment of the three groups showed that the TPD of the BBS group was larger than that of the HB group (*P* < 0.05), and LVEF in the HB group and the BBS group had significant difference as compared with the BO group. There was no significant difference in LVEF between the BBS group and HB group (*P* > 0.05)

After 3 months post-MI, all the survival animals showed a significant perfusion defect in the apical level and anterior and anterior septal of mid-level in the left ventricle (Fig. 7a). The TPD in the BO group, HB embolism group, and BBS group were 18.5 ± 1.0, 35.4 ± 1.5, and 39.7 ± 1.2 %, respectively. There were significant differences in all the three groups. Especially, the TPD of the BBS group was larger than the HB group (*P* < 0.05, *n* = 10) (Fig. 7b, c). In addition, the LVEF in the three groups were 50.5 ± 1.6, 43.5 ± 2.2, and 43.0 ± 2.4 %, respectively. LVEF in the HB group and BBS group calculated from SPECT data had significant differences as compared with the BO group, which is similar to the results from echocardiogram. There appeared no significant difference in ejection fraction between the BBS group and HB group (*P* > 0.05) (Fig. 7d).

### Larger infarct size in embolism groups than the BO group

**Fig. 8:**
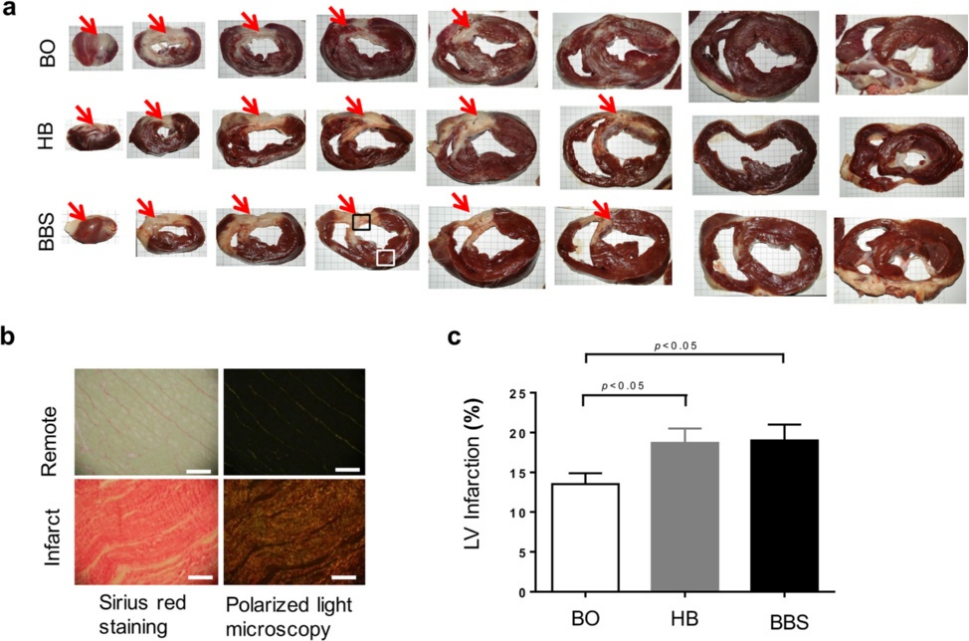
Larger infarct size in embolism groups than the BO group. **a** TTC staining of serial short axis section confirmed the formation of myocardial infarction at anterior septal and anterior wall myocardium. The *red arrows* indicate the infarct zone (the interval of coordinate paper is 5 mm). **b** The tissue from the infarct zone (*black box*) was further confirmed as proliferation of fibrous tissue compared with remote zone (*white box*) by Sirius red staining and polarized light observation. Scale bars = 50 rom. **c** Semi-quantitative assessment of infarct size in the three groups

Infarct patterns of these three groups were shown by representative triphenyl tetrazolium chloride (TTC) staining (Fig. 8a). The proliferation of fibrous tissue in the infarct zone was further confirmed by Picro Sirius red staining and polarized light observation (Fig. 8b). The infarct size (expressed as LV infarction, %) in the BO group, HB group, and BBS group were 13.5 ± 1.4, 18.7 ± 1.8, and 19.0 ± 2.0 %, respectively. The HB group and BBS group witnessed significant difference compared with the BO group (*P* < 0.05). There was no significant difference between the two embolism groups (*P* > 0.05) (Fig. 8c).

## Discussion

Porcine MI model has gained popularity due to greater similarity in coronary anatomy and cardiac physiology with humans [28]. Especially, minipig is an ideal experimental animal with smaller body size and genetic stability. In the present study, we set up a new convenient and effective method for chronic LAD embolization by BBS in minipigs. In a previous study, Reffelmann et al. and Dariolli et al. [14, 17] had proposed that the sterile sponge can be advanced directly into coronary artery by a balloon catheter to induce MI. However, the sponge is easy to shift and also be off load from the guidewire because of the fusiform structure of the balloon tip. In our study, immediate satisfactory results can be achieved when target vessel was embolized by half-balloon and subsequent thrombosis. However, we found that even though immediate results were satisfactory, LAD recanalization was high during the follow-up. This recovered forward blood flow will lead to misevaluation for the effectiveness of subsequent interventions in the animal model. According to our results, the myocardial perfusion in the BBS group is poorer than that in the HB group at 3 months post-MI, which may be related to the recanalization in the HB group. Meanwhile, other methods did not detect the difference between the groups.

In the BBS group with sequential embolism, the metal marker of the balloon can be used to precisely locate the embolism position to keep the consistent infarct size. Subsequent thrombus gradually formed, and the blood flow occluded totally within about 10–20 mins, which can be considered as ischemic preconditioning to protect from acute myocardial injury. This might be the reason for the significant reduction of VF caused by sudden and complete blockage of blood flow. In addition, the second free half-balloon, sponge, and half-balloon with catheter formed a sandwich-like structure, which can be pushed easily to the target position. This sequential embolization method maximized the effectiveness and achieved the purpose of complete and permanent occlusion. Meanwhile, the BBS group did not significantly increase the operation time and incidence of VF, which made the chronic MI model more conducive to establish.

As for the evaluation for the chronic MI, heart function, and left ventricular remodeling, several imaging modalities including echocardiography, SPECT/CT, and PET/CT were applied in preclinical study [26, 27]. However, each imaging technique holds its strength and weakness. In order to fully evaluate the whole aspect of the infarcted heart, we applied cardioangiography, echocardiography, SPECT/CT, and ^18^F-FDG PET/CT. Data showed that the infarct size and embolized artery were more stable and repeatable in the BBS group than that in other groups. As a result, the chronic MI model is more consistent and conducive as compared with others. Furthermore, due to the shorter operation time than conventional balloon blocking, the minipig could better tolerate operation. Subsequently, animals were given oxygen inhalation with the nasal catheter and without the tracheal intubation during the operation, which made the experiment simpler and more convenient.

Although this methodology appears to have significant utility for animal research in chronic myocardial infarctions, it is recognized that several study limitations exist. Due to the consideration of experimental cost, the group of 60-min balloon occlusion was established as the only conventional control group, and ^18^F-FDG PET/CT myocardial metabolism imaging was only used in the BBS group to verify the formation of myocardial infarction.

## Conclusions

Compared to the conventional modeling induced by balloon blocking, the method of sequential balloons-sponge embolization could induce myocardial infarction with consistent and sustained embolization and gain higher operation success rate and better repeatability in minipigs, which holds a promising method for preclinical myocardial infarction study.

## Additional files

Additional file 1: Classic methods have been fully described by previous investigators and introduced. (DOCX 29 kb) Additional file 2: Left ventricular angiography of pre-MI in BBS group. (AVI 2406 kb) Additional file 3: Left ventricular angiography of 3 months post-MI in BBS group. (AVI 2427 kb)
