# Supplementary material for: Preclinical modeling and multimodality imaging of chronic myocardial infarction in minipigs induced by novel interventional embolization technique
Source: EJNMMI Res. 2016 Jul 8;6:59. doi: 10.1186/s13550-016-0214-7 (PMC4938836; doi:10.1186/s13550-016-0214-7)
Supplement: Additional file 1: — Classic methods have been fully described by previous investigators and introduced. (DOCX 29 kb) [file 13550_2016_214_MOESM1_ESM.docx]

**SUPPLEMENTAL MATERIAL**

**Detailed Methods**

**Electrocardiogram and echocardiogram**

Twelve-lead ECG was recorded under anesthesia. Two-dimensional (2D) and M-mode UCG assessment were performed at the parasternal location according to standard imaging techniques with a VIVID 7 ultrasound imaging system (GE, New York, USA). Left ventricular ejection fraction (LVEF) and end-diastolic volumes (LVEDV) were determined as previously described[[1](#_ENREF_1)]. Three consecutive heart cycles were measured for each minipig and all measurements averaged. All images were recorded and analyzed offline and the results were determined by the consensus of two experienced ultrasound technicians blinded to the treatment assignment.

**Quantitative cardiac gated SPECT/CT imaging**

ECG-gated Single Photon Emission Computed Tomography/Computed Tomography (SPECT/CT) imaging was performed with a system (SymbiaT2, Siemens, Germany). The anaesthetized animals were placed in the right lateral decubitus position for the imaging. The imaging was assessed at a dose of 0.3 mCi/kg 99mTc-sestamibi (99mTc-MIBI) via the ear vein. CT images were acquired with the 2-slice CT component of the SPECT/CT scanner. Myocardial perfusion images were acquired 60 min after 99mTc-MIBI injection using a dual-head camera with high-resolution collimators. The camera energy window (20%) was set on the 140 keV photopeak of 99mTc-MIBI. Thirty-two images (64 × 64 matrix) were acquired for 30 seconds each with 180° rotation. The tomograms were reconstructed in the vertical and horizontal long-axis and short-axis planes. A quantitative gated SPECT (QGS, Cedars Cardiac Quantification) software was used for processing of all recordings and semi-quantitative assessment of LV volumes and perfusion defect. Image fusion of SPECT and CT was performed with a processing station (SyngoMI VA30A, SIEMENS, Germany). Myocardial perfusion images were acquired and semi-quantitative assessment of LVEF and total perfusion deficit (TPD) were processed as described in a previous study [[2](#_ENREF_2)].Two experienced nuclear medicine technicians who were blinded to the treatment allocation processed all the recordings.

**^18^F-FDG PET/CT myocardial metabolism imaging**

^18^F-FDG Positron Emission Tomography/ Computed Tomography(PET/CT) imaging was performed with an available system (Biograph40, Siemens). Animals were sedated with the intravenous injection of ketamine and placed at the right lateral decubitus position. The imaging was assessed at a dose of 0.15 mCi/kg ^18^F-FDG via the ear vein. Myocardial metabolism images were acquired 40 mins after ^18^F-FDG injection using high-resolution collimators. The fusion of PET and CT images was performed on a processing station (SyngoMI software; Siemens, Germany)[[3-6](#_ENREF_3)]. Two experienced nuclear medicine technicians who were blinded to the treatment allocation processed all the recordings.

**Histological analysis**

Minipigs were sacrificed with 10% potassium chloride solution of 20 ml at 3 months post-MI for histological analysis. Cardiac tissues were harvested and sectioned into 10-mm-thick cross-sectional myocardial slices. Serial short axis myocardial sections were stained for 20 min with 1% 2, 3, 5-triphenyltetrazoliumchloride (TTC) at 37° to delineate infarct borders. The digital images were taken and measured with an interactive computerized image analysis system (Optimas 5.2 color image analysis; Germany). Myocardial infarct size was expressed as a percentage of infarct area over the total left ventricle (LV) area[[3](#_ENREF_3), [7](#_ENREF_7)]. Picro Sirius red stain was used to highlight collagen deposition associated with post-MI fibrosis.

**References**

1. Weidemann F, Dommke C, Bijnens B, Claus P, D'Hooge J, Mertens P et al. Defining the transmurality of a chronic myocardial infarction by ultrasonic strain-rate imaging: implications for identifying intramural viability: an experimental study. Circulation. 2003;107(6):883-8.

2. Tao B, Cui M, Wang C, Ma S, Wu F, Yi F et al. Percutaneous intramyocardial delivery of mesenchymal stem cells induces superior improvement in regional left ventricular function compared with bone marrow mononuclear cells in porcine myocardial infarcted heart. Theranostics. 2015;5(2):196-205. doi:10.7150/thno.7976.

3. Lautamaki R, Schuleri KH, Sasano T, Javadi MS, Youssef A, Merrill J et al. Integration of infarct size, tissue perfusion, and metabolism by hybrid cardiac positron emission tomography/computed tomography: evaluation in a porcine model of myocardial infarction. Circulation Cardiovascular imaging. 2009;2(4):299-305. doi:10.1161/circimaging.108.846253.

4. Schuleri KH, Boyle AJ, Centola M, Amado LC, Evers R, Zimmet JM et al. The adult Gottingen minipig as a model for chronic heart failure after myocardial infarction: focus on cardiovascular imaging and regenerative therapies. Comparative medicine. 2008;58(6):568-79.

5. Positron emission tomography for the assessment of myocardial viability: an evidence-based analysis. Ontario health technology assessment series. 2010;10(16):1-80.

6. Single photon emission computed tomography for the diagnosis of coronary artery disease: an evidence-based analysis. Ontario health technology assessment series. 2010;10(8):1-64.

7. Gao E, Lei YH, Shang X, Huang ZM, Zuo L, Boucher M et al. A novel and efficient model of coronary artery ligation and myocardial infarction in the mouse. Circulation research. 2010;107(12):1445-53. doi:10.1161/circresaha.110.223925.
